# Association between time to stent dysfunction and the anti-tumour effect of systemic chemotherapy following stent placement in patients with pancreaticobiliary cancers and malignant gastric outlet obstruction: a retrospective cohort study

**DOI:** 10.1186/s12885-021-08336-z

**Published:** 2021-05-19

**Authors:** Satoshi Kobayashi, Makoto Ueno, Shuhei Nagashima, Yusuke Sano, Kuniyuki Kawano, Taito Fukushima, Hiroyuki Asama, Shun Tezuka, Manabu Morimoto

**Affiliations:** Department of Gastroenterology, Hepatobiliary and Pancreatic Medical Oncology Division, Kanagawa Cancer Center, 2-3-2, Nakao, Asahi-ku, Yokohama City, 241-0815 Japan

**Keywords:** Duodenal stenting, Systemic chemotherapy, Cancer, Stent dysfunction, Response

## Abstract

**Background:**

Malignant gastric outlet obstruction (MGOO) occasionally occurs due to pancreaticobiliary cancer. Endoscopic duodenal stenting (DS) is a common treatment for MGOO. However, it has been reported that DS does not have sufficient patency time for it to be used in patients who have a potentially increased lifespan. Nowadays, systemic chemotherapy for pancreaticobiliary cancer has developed, and its anti-tumour effect would make time to stent dysfunction longer.

Therefore, we retrospectively evaluated the association between objective response to systemic chemotherapy, followed by DS and time to stent dysfunction in patients with advanced pancreaticobiliary cancer.

**Methods:**

This retrospective study included 109 patients with advanced pancreaticobiliary cancer who received systemic chemotherapy after DS. Patients who showed complete or partial response were defined as responders. The rest were defined as non-responders. Time to stent dysfunction was compared between responders and non-responders using the landmark analysis at 2 months after DS. Death without recurrence of MGOO was considered as a competing risk for time to stent dysfunction.

**Results:**

Combination and monotherapy regimens were adopted for 46 and 63 patients, respectively. Median progression-free survival and overall survival were 3.2 months (95% confidence interval [CI], 2.4–4.0) and 6.0 months (95% CI, 4.6–7.3). Objective response was observed in 21 patients (19.3%). Median time to stent dysfunction was 12.5 months (95% CI, 8.4–16.5) in the entire cohort. In 89 patients, responders had a lower cumulative incidence of stent dysfunction than non-responders: 9.5 and 19.1% at 6 months, and 19.0 and 27.9% at 1-year, respectively. There was difference of time to stent dysfunction between responders and non-responders among patients who received combination regimen as the first-line treatment with *p*-value of 0.009: cumulative incidence was 0 and 42.9% at 6 months, and 9.3 and 57.1% at 1-year, respectively.

**Conclusions:**

Longer time to stent dysfunction is expected when systemic chemotherapy following DS suppresses tumour progression; DS is slated to be a standard treatment for MGOO even in patients with pancreaticobiliary cancer and a long lifespan.

**Supplementary Information:**

The online version contains supplementary material available at 10.1186/s12885-021-08336-z.

## Background

Pancreatic cancer is the third leading cause of cancer-related death in the United States; death due to this disease accounted for approximately 43,000 cases in 2017 [1]. It was the fourth leading cause of cancer death in Japan in 2018 [2]. Biliary tract cancer includes malignant cancer originating from the extrahepatic and hilar bile ducts, gallbladder, and ampulla of Vater. It is a relatively rare cancer in the United States or Europe; however, it is more common in East Asian countries, accounting for 18,000 cases in Japan. As the disease progresses, the primary tumour grows up; malignant gastric outlet obstruction (MGOO) due to tumour invasion through the layers of the gastroduodenal wall occurs in approximately 10–20% of patients with pancreaticobiliary cancer [3–5]. Gastric outlet obstruction is a crucial issue for patients with these cancers because it induces nausea, vomiting, and anorexia, which can result in life-threatening comorbidities.

Gastrojejunostomy (GJ) has been widely used as a palliative treatment option for MGOO; in the early 1990s, duodenal stenting (DS) was first developed and reported as an alternative procedure to GJ [6]. According to the reviews of some clinical trials, DS is especially recommended for patients with MGOO who had a life expectancy of less than 3 months since the patency of DS is approximately 3 months, and additional interventions are often required [7–9].

Advanced pancreaticobiliary cancer has a poor prognosis, even in patients who received systemic chemotherapies after resolving MGOO, since systemic chemotherapies (such as gemcitabine (GEM) monotherapy) had little efficacy in prolonging patient survival and a low response rate [10, 11]. Patients with advanced pancreaticobiliary cancer were candidates for DS, even if they could receive subsequent chemotherapy after resolving MGOO. We evaluated the clinical effectiveness and safety of DS for patients with pancreatic cancer [12]. We concluded that DS was an effective treatment for patients with advanced pancreatic cancer and MGOO in terms of its safety and smooth performance of subsequent chemotherapies.

In recent years, systemic chemotherapies have been developed for advanced pancreaticobiliary cancer, such as FOLFIRINOX (fluorouracil, leucovorin, irinotecan and oxaliplatin) [13] and GEM plus nab-paclitaxel [14] for pancreatic cancer, and GEM plus cisplatin [15] and GEM plus S-1 for biliary tract cancer [16]. Response rate for these regimens has been reported as 30–60%; overall survival was 10–11 months [17]. Based on these data, patients who were candidates for these regimens will be suitable for GJ rather than for DS, as the treatment option for MGOO; however, DS can be another option if chemotherapy can prolong the patency of DS. Therefore, in this study, we aimed to assess the association between cumulative incidence of stent dysfunction for DS and the efficacy of systemic chemotherapy.

## Methods

### Patients

This was a retrospective cohort study. We reviewed the medical records of 317 consecutive patients with advanced pancreaticobiliary cancer who underwent DS for MGOO at our institution between July 2010 and December 2019. Patients enrolled in this study were also provided with the opportunity to opt-out of having any information published.

### Duodenal stenting

We considered patients as candidates for endoscopic DS if they met the following criteria as described in our previous report [12]: 1) unresectable or recurrent disease that could not be cured with surgical resection; 2) histologically or cytologically proven pancreaticobiliary cancer; and 3) MGOO due to a stricture in the stomach or duodenum that was confirmed through radiological or endoscopic findings. Duodenal stent placement was contraindicated for patients who met the following conditions: 1) small bowel strictures or functional disorder induced by peritoneal dissemination; 2) stent placement risk factors due to haemorrhagic status or cardiopulmonary problems; 3) life expectancy of fewer than 2 weeks. We used several types of stents, such as the WallFlex or WallFlex Duodenal Soft (Boston Scientific Corporation, Marlborough, MA, USA) (22 mm with a proximal flare of 24 mm in diameter); Niti-S D pyloric/duodenal, and Niti-S COMVI™ Pyloric duodenal (Taewoong Medical Co., Ltd., Seoul, Korea) (22 mm in diameter without proximal flares); HANAROSTENT® Naturfit™ Duo (22 mm in diameter without proximal flares) (Boston Scientific Corporation, Marlborough, MA, USA). Stent lengths vary from 60 to 120 mm; we selected the stent according to the stricture length. The Niti-S COMVI™ was only used for patients who already had a tumour haemorrhage because it is a covered stent. Uncovered stents were used at the physician’s discretion: the WallFlex Duodenal Soft and HANAROSTENT could be used after August 2017 and May 2019; the other stents could be used throughout the entire period of this study. Stent placement was performed under sedation; the stricture was mainly identified endoscopically. In cases where the stricture was located at the distal end of the horizontal portion of the duodenum, we performed fluoroscopic duodenography using a contrast medium (Gastrografin Oral Enema, Bayer HealthCare Pharmaceuticals, Leverkusen, Germany) to identify the stricture. A 0.035-in. guidewire (Hydra Jagwire, Boston Scientific Corporation) was inserted through the stricture, and the duodenal stent was positioned across it under fluoroscopic guidance. Stent length was chosen according to the stricture length and the position of the pylorus/ampulla of Vater. Finally, the stent was deployed, and its patency was confirmed by injection of the contrast medium. Types of DSs used with each patient depended on the physician’s discretion and their availability. After stent placement, patients could resume consuming liquids on day 1, soft foods on day 2, and solid foods on day 3, as long as no symptoms of MGOO exacerbation were observed. In cases of MGOO recurrence, a second/third duodenal stent was placed using the stent-in-stent technique.

### Systemic chemotherapy after duodenal stenting

We considered applying systemic chemotherapy after duodenal stenting for patients who met the following criteria: 1) Eastern Cooperative Oncology Group performance status (ECOG PS) of 0–2; 2) achievement of full oral intake; 3) adequate liver and kidney functions; 4) preserved bone marrow function: neutrophil count > 1500/μL; platelet count > 100,000/μL; and 5) a life expectancy of at least 3 months. We chose the chemotherapy regimens according to each patient’s consent and conditions, as follows: e.g., patients who had ECOG PS of 0–1 and were aged < 75 years were introduced to combination chemotherapeutic regimens, such as modified FOLFIRINOX [18] or GEM plus nab-paclitaxel for pancreatic cancer, or GEM plus cisplatin or GEM plus S-1 for biliary tract cancer. Patients who were ineligible for these combination regimens (but eligible for less toxic therapies) were introduced to monotherapy regimens, such as GEM alone or S-1 alone. We adopted these regimens not only as first-, but also as second- or third-line treatments. The initial dose of each agent was reduced at the physician’s discretion. Chemotherapy continued until disease progression, intolerable adverse events, or patient refusal.

### Clinical outcomes

The clinical symptom was evaluated according to the GOO scoring system [4]: score 0, no oral intake; score 1, liquids only; score 2, soft foods; score 3, solid foods/full diet. Stent dysfunction was defined as the recurrent symptoms of GOO due to tumour ingrowth and overgrowth, stent migration, and food impaction, which was confirmed based on radiological and/or endoscopic findings and not on symptoms such as nausea or vomiting. Time to stent dysfunction (TTSD) was determined starting from the date of DS through the date of diagnosis of stent dysfunction. Tumour ingrowth and overgrowth, stent migration, and food impaction were considered stent dysfunction-related events. Patients who could not eat orally due to disease progression were treated as a competing risk, and those who could eat orally at the last follow-up were treated as censored cases for the evaluation of TTSD. Additionally, we complementarily evaluated time to impossible oral food intake by considering the impossibility of oral intake due to disease progression as well as stent dysfunction. Overall survival and progression-free survival were determined starting on the date of initiation of systemic chemotherapy through the date of documented disease progression or any cause of death and the date of death due to any cause or the last follow-up, respectively. The best response during chemotherapy was radiologically evaluated according to the Response Evaluation Criteria in Solid Tumors version 1.1 [19].

### Statistical analysis

Results are expressed as median and were analysed using the SPSS Statistics 23 software (IBM SPSS, Inc., Chicago, IL, USA). Changes in the MGOO score [4] before and after duodenal stent placement were evaluated using the Wilcoxon signed-rank test. We calculated overall survival and progression-free survival using the Kaplan-Meier method; we used the log-rank test to compare these time-to-event parameters in two groups. Cumulative incidence of stent dysfunction and impossible oral food intake were expressed with 6-months and 1-year rate; we used the Gray test to compare these time-to-event parameters with competing risk in two groups. Patients were divided into two groups according to the objective response to chemotherapy. Patients who showed complete or partial responses were defined as responders, and those who showed stable or progressive disease were defined as non-responders. We compared the cumulative incidence of stent dysfunction of responders with that of non-responders by conducting a landmark analysis (landmark at 2 months after DS) to avoid guarantee-time bias. The date of the last follow-up was 21 July 2020, and we fixed the data on 06 August 2020.

## Results

### Patient characteristics

**Fig. 1:**
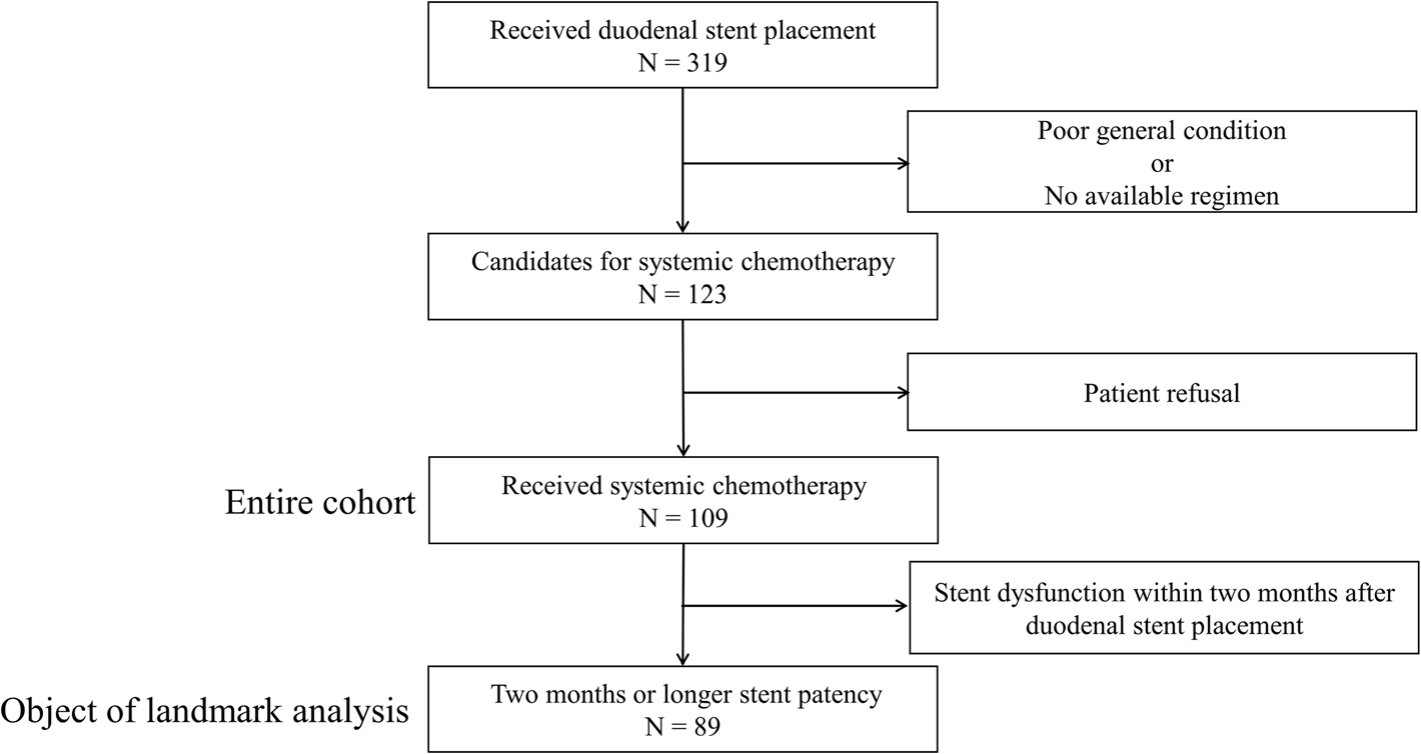
CONSORT flow diagram of the study. Of 319 patients who received duodenal stent placement for malignant gastric outlet obstruction due to advanced pancreaticobiliary cancer, 109 received systemic chemotherapy followed by duodenal stent placement, and 89 had two months or longer to stent dysfunction

Of 319 consecutive patients who had undergone DS, the study included 109 patients who received systemic chemotherapy after DS (Fig. 1). Patient characteristics are shown in Table 1. Primary diseases were pancreatic adenocarcinoma, biliary tract cancer, pancreatic grade 2 neuroendocrine neoplasm (G2 pNEN), and pancreatic neuroendocrine carcinoma (pNEC) in 90, 15, 3, and 1 patient, respectively. The median age of the enrolled patients was 68 years (range: 31–81); 85 patients (77%) had metastatic cancer, and 52 of them had peritoneal dissemination.

**Table 1:** Baseline characteristics of patients at the initiation of systemic chemotherapy following duodenal stent placement

Characteristics	
Age (years), median (median)	68.0 (31–81)
Sex, n (%)	
Male	54 (50)
Female	55 (50)
Primary disease, n (%)	
Pancreatic adenocarcinoma	90 (83)
Biliary tract adenocarcinoma	15 (14)
Pancreatic neuroendocrine neoplasms, Grade 2	3 (3)
Pancreatic neuroendocrine carcinoma	1 (1)
Disease status, n (%)	
Locally advanced	24 (22)
Metastatic	85 (78)
ECOG^*^ performance status	
0	18 (17)
1	62 (57)
2	29 (27)
Position of bowel stricture, n (%)	
Oral side of the papilla of Vater	33 (30)
Across the papilla of Vater	26 (24)
Anal side of the papilla of Vater	50 (46)
Duodenal stent, n (%)	
WallFlex Duodenal	46 (42)
WallFlex Duodenal Soft	3 (3)
Niti-S D type	46 (42)
Niti-S COMVI™	4 (4)
HANAROSTENT® Naturfit™ Duo	10 (9)
Number of chemotherapy regimens prior to duodenal stenting, n (%)	
0	47 (43)
1	49 (45)
2–3	13 (12)
^¶^CRP (mg/dL), median (range)	0.79 (0.05–12.9)
Distribution, n (%)	
< 1.0 mg/dL	60 (55)
≥ 1.0 mg/dL	49 (45)
Albumin (g/dL), median (range)	3.3 (2.1–4.1)
Distribution, n (%)	
< 3.5 mg/dL	67 (62)
≥ 3.5 mg/dL	42 (39)
^#^CA19–9 (U/mL), median (range)	928.9 (0–408,800.0)
Distribution, n (%)	
< 1000 U/mL	55 (50)
≥ 1000 U/mL	54 (50)

^*^ECOG Eastern Cooperative Oncology Group, ^¶^*CRP* C-reactive protein, ^#^*CA19–9* carbohydrate antigen 19–9

### Duodenal stent placement

The duodenal stents used were WallFlex, Niti-S D type, HANAROSTENT® Naturfit™ Duo, Niti-S COMVI™, and WallFlex Soft in 46, 46, 10, 4, and 3 patients, respectively. Strictures were located on the oral side, across, and anal side of the papilla of Vater in 33, 26 and 50 patients, respectively. Among those, 6 patients with a MGOO score of 3 required DS: MGOO on the horizontal part of the duodenum was complicated with cholangitis in 3 patients; 2 patients had strictures in both the duodenal bulb and common bile duct; hence, DS was required before endoscopic retrograde biliary stenting. The MGOO score was better after DS compared to that before it: the average value of GOOSS was 1.1 and 3.0, respectively, with a *p*-value < 0.001. The median time required to tolerate food intake was just 1 day (range: 1–18 days).

### Systemic chemotherapy

**Fig. 2:**
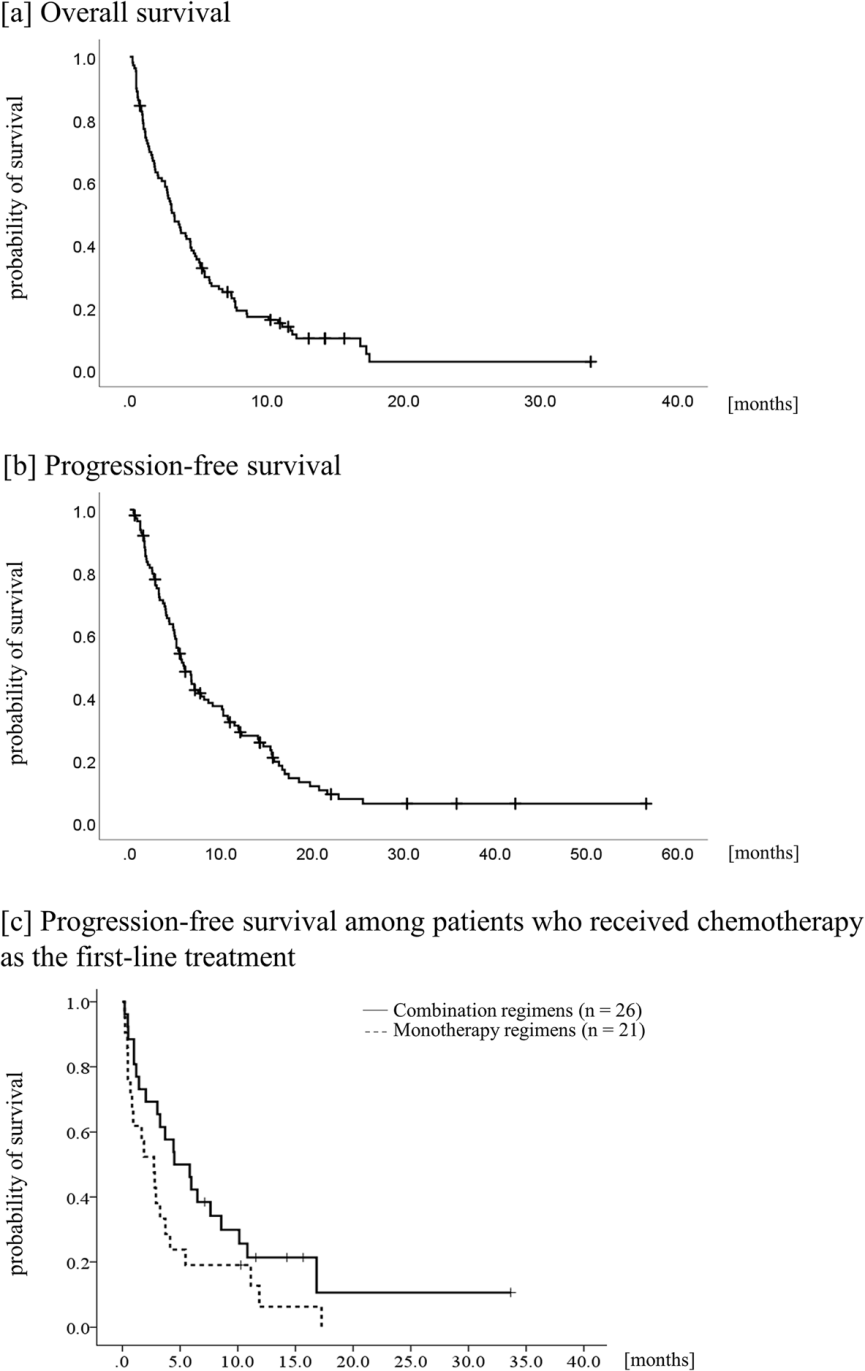
Kaplan-Meier curve of (**a**) overall survival and (**b**) progression-free survival. In the entire cohort, which consisted of 109 patients, median progression-free survival and overall survival rates after duodenal stent placement was 3.2 months (95% confidence interval, 2.4–4.0) and 6.0 months (95% confidence interval, 4.6–7.3), respectively. **c** Comparison of 26 patients who received combination regimen (solid line) and 21 patients who received a monotherapy regimen (dotted line) as the first-line treatment, median progression-free survival rates were 4.5 months (95% confidence interval, 1.7–7.3) and 2.7 months (95% confidence interval, 1.0–4.4) respectively, with a *p*-value of 0.075

The median time from the date of stent placement through the date of initiation of chemotherapy was 12 (range: 1–60) days. The detailed administered regimens are shown in supplement Table 1. Combination regimens were adopted as the first-, second-, third- and fourth-line treatment for 26, 13, 6 and 1 patient and monotherapy regimens as first-, second and third-line treatment for 21, 15 and 6 patients, respectively. The median progression-free survival and overall survival were 3.2 months (95% CI, 2.4–4.0) and 6.0 months (95% CI, 4.6–7.3) (Fig. 2a and b). Regarding the objective response, partial response (PR), stable disease (SD), and progressive disease (PD) were observed in 21, 45, and 43 patients, respectively. Response and disease control rates were 19.2 and 60.6%, respectively. Patients who received combination regimens had longer progression-free survival and higher response rates than those who received monotherapy regimens in the first-line setting; progression-free survival was 4.5 months (95% CI, 1.7–7.3) and 2.7 months (95% CI, 1.0–4.4) with a *p*-value of 0.075 (Fig. 2c), and response rates were 46.2 and 4.8% with a *p*-value 0.002, respectively.

### Time to duodenal stent dysfunction

**Fig. 3:**
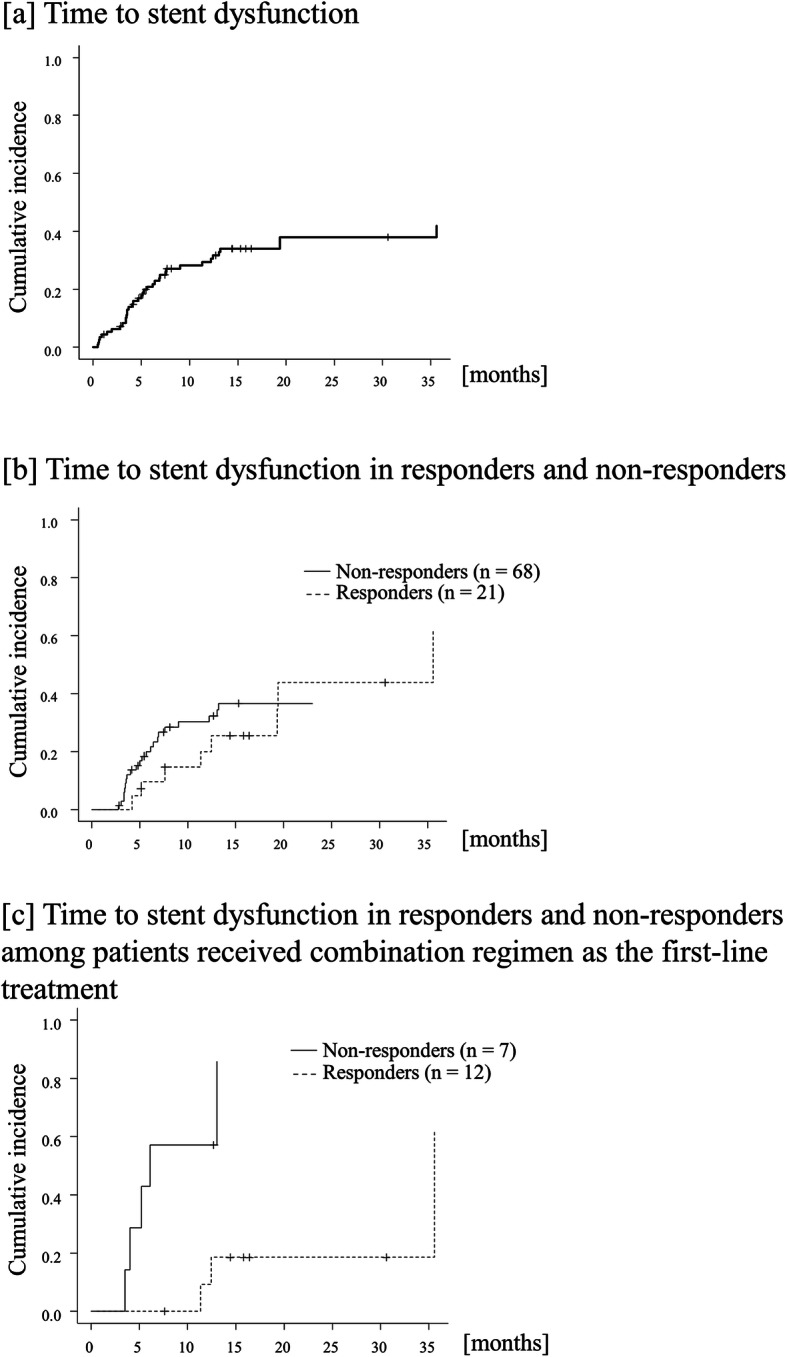
Time to stent dysfunction. **a** Entire cohort. The median time to stent dysfunction was not reached (95% CI, 35.6-not applicable) and cumulative incidence of stent dysfunction was 17.5 and 27.9% at 6-months and 1-year, respectively; **b** Comparison between patients who showed a response (dotted line) and those who did not (solid line). Cumulative incidence of stent dysfunction in responders (*n* = 21) and in non-responders (*n* = 68) was 9.5 and 19.1% at 6 months, and 19.0 and 27.9% at 1-year, respectively (*p*-value = 0.69); **c** Comparison between responders (dotted line) and non-responders (solid line) among patients who received a combination regimen as the first-line treatment. Cumulative incidence of stent dysfunction in responders (*n* = 12) and in non-responders (*n* = 7) was 0 and 42.9% at 6 months, and 8.3 and 57.1% at 1-year, respectively (*p*-value = 0.009)

During the median observation time of 4.8 (range: 0.5–35.6) months, stent dysfunction was observed in 38 patients (35%), and the impossibility of oral intake due to disease progression was observed in 53 (49%); the remaining 19 patients were censored at the time of analysis. Of these, stent migration was observed in one patient; in the remaining patients, the reason for stent dysfunction was stent occlusion. Cumulative incidence of stent dysfunction was 17.5 and 27.9% at 6-months and 1-year (Fig. 3a). Cumulative incidence of impossible oral food intake at 6-months and 1-year was 55.0 and 71.6% in the entire cohort, and 42.3 and 53.8% in patients who received combination regimens as the first-line treatment, respectively (Supplement Figure 1).

Landmark analysis at the 2-month landmark point revealed that cumulative incidence of stent dysfunction was lower in responders (*n* = 21) and in non-responders (*n* = 68) with a *p*-value of 0.69: cumulative incidence was 9.5 and 20.1% at 6 months, and 20.1 and 30.3% at 1-year, respectively (Fig. 3b and Table 2). There were differences in cumulative incidence between responders and non-responders in patients who received a combination regimen as the first-line treatment with *p*-value of 0.009 (Fig. 3c): cumulative incidence was 0 and 42.9% at 6 months, and 9.3 and 57.1% at 1-year, respectively. Time to impossible oral food intake was longer in responders than in non-responders with a p-value < 0.001: cumulative incidence was 19.0 and 57.3% at 6 months, and 39.7 and 82.6% at 1-year, respectively (supplement Figure 2).

**Table 2:** Cumulative incidence of stent dysfunction in each background

Factor	at 6-months, %	at 1-year, %	*p*-value^†^
Primary disease			0.33
Pancreatic adenocarcinoma	21.6	31.7	
Biliary tract cancers	13.9	13.9	
Pancreatic neuroendocrine tumours	37.5	37.5	
Disease status			0.92
Locally advanced	22.1	31.4	
Metastatic	20.6	28.9	
^*^ECOG PS			0.37
0–1	19.3	27.7	
2–3	24.6	33.6	
Chemotherapy regimen			0.61
^¶^Combo	23.0	28.4	
^#^Mono-	19.4	30.3	
Indication of chemotherapy			0.74
First-line	21.6	28.3	
Salvage-line	20.5	30.7	
Response to chemotherapy^a^			0.69
Partial response	9.5	20.1	
Stable or progressive disease	20.1	30.3	

^a^ According to the New response evaluation criteria in solid tumours, version 1.1

^*^ECOG PS Eastern Cooperative Oncology Group performance status, ^¶^*Combo* combination regimens such as FOLFIRINOX, gemcitabine plus nab-paclitaxel, gemcitabine plus cisplatin and gemcitabine plus S-1, ^#^*Mono* monotherapy such as gemcitabine alone or S-1 alone

^†^ The Gray test

## Discussion

According to previous studies, DS is inferior to GJ in terms of TTSD. However, DS is superior to GJ in terms of safety, rapid symptom relief, and shorter time required to resume food intake. Based on the results, treatment recommendations for pancreatic cancer by the National Comprehensive Cancer Network and Japanese Pancreas Society state that proper use of the two treatment options may depend on the patients’ prognostic estimates [20, 21]. These recommendations do not state which is better for patients who will receive chemotherapy; their references date from 2000 to 2010, when the response rate of systemic chemotherapy was dismal. In recent decades, systemic chemotherapy for advanced pancreaticobiliary cancer has developed. Therefore, we evaluated the influence of chemotherapy on stent dysfunction in patients who underwent DS and subsequent chemotherapy in 2010–2018. Our results showed that TTSD after DS was prolonged after chemotherapy through tumour shrinkage, especially when using a combination regimen as the first-line treatment. Therefore, DS could be a good treatment option, not only for patients with short life expectancy but also for those who are eligible for a combination regimen and can expect an increased life expectancy. In other words, DS can be applied to all patients with unresectable pancreaticobiliary cancer as a standard treatment option for MGOO, along with advances in chemotherapy.

There are several reports on the effect of chemotherapy on TTSD of the biliary stent; some studies have concluded that chemotherapy decreased TTSD by inducing bacterial colonisation in the bile ducts through its side effect of immunosuppression [22]; others have stated opposite conclusions, speculating that positive effect via tumour control would compensate for its negative effect in patients who received chemotherapy [23]. Regarding DS, the main causes of stent dysfunction are tumour growth and food impaction. Bacterial colonisation does not influence its patency. Therefore, we believe this might be why chemotherapy has positive effects on the TTSD of DS. Kim et al. have also reported that time to progression was an independent protective factor against restenosis of DS in patients with gastric cancer [24]. When that report was published, the response rate and time to progression for unresectable gastric cancer was around 30–40% and 5–7 months, respectively [25, 26]. These values were close to those of recent standard chemotherapy for advanced pancreaticobiliary cancer [13–16]. Patients who received combination regimens as the first-line treatment had a median progression-free survival of 4.5 months and a response rate of 46.2% in our study. Therefore, we think this was another reason why our study could show the association between TTSD for DS and the effects of chemotherapy.

Overall survival was 4.6 months or longer in 95% of the patients included in our cohort. According to this review article [7], surgical bypass is recommended for patients with a life expectancy of 3 months or longer; most patients had been eligible for surgical bypass rather than for DS. However, in this study, TTSD for DS was long enough that the patients survived without recurrent MGOO; cumulative incidence of stent dysfunction was 17.5% at 6-months and 27.9% at 1-year. Based on the results, we believe that DS could also be recommended as a treatment option for MGOO for patients who are scheduled to receive intensive first-line chemotherapy for unresectable pancreaticobiliary cancer. Development of systemic chemotherapy for these diseases is awaited; DS will have longer TTSD in the future than currently, along with the improvement in response rate and progression-free survival in the future. Regarding safety, DS is considered a better option than surgical bypass unless complications included stent dysfunction [7, 9]. Although we did not compare DS with the surgical bypass in this study, we believe that DS is neither inferior to surgical bypass in terms of efficacy nor superior in terms of safety.

The existing definition of stent dysfunction has affected the results, as the majority of patients could not eat orally due to disease progression; their stents were found to be patent, even in the final phase of the life. Regarding biliary stenting, the TOKYO criteria (2014) for transpapillary biliary stenting recommend patient death and complications other than recurrent biliary obstruction requiring stent removal be treated as censored cases at the time of death or stent removal, respectively [27]. According to the criteria mentioned above, our primary definition of stent dysfunction was set as recurrent MGOO with stent thrombosis or migration. Primary data analysis showed that stent dysfunction was only observed in one-third of patients. The small number of events will lower the statistical power to detect differences; hence, we conducted a supplementary analysis using disease progression as an event to support the robustness of the primary analysis.

This study has some limitations. Its small number of patients and retrospective design is likely to cause several biases. Our study did not have a surgical bypass cohort; therefore, a direct comparison between DS and surgical bypass was impossible. Indications for each chemotherapy regimen depended on the physician’s discretion. In addition, patients and regimens in this study formed a complex combination of several diseases and regimens.

## Conclusions

Despite these limitations, we concluded that the anti-tumour effects of systemic chemotherapy improve TTSD of DS in pancreaticobiliary cancers; DS could be preferable not only for patients who have a life expectancy of fewer than 3 months but also for those who are scheduled to receive systemic chemotherapy and are slated to have a longer life expectancy. Therefore, DS can be the standard treatment option for MGOO in unresectable pancreaticobiliary cancers.

## Supplementary Information

**Additional file 1: Supplement Figure 1.** Time to impossible oral food intake in the entire cohort (solid line) and patients who received combination regimens as the first-line treatment (dotted line). Cumulative incidence of impossible oral food intake at 6-months and 1-year was 55.0 and 71.6% in the entire cohort, and 42.3 and 53.8% in patients who received combination regimens as the first-line treatment, respectively.

**Additional file 2: Supplement Figure 2.** Time to impossible oral intake food in responders (dotted line) and non-responders (solid line) among patients who received combination regimen as the first-line treatment. Time to impossible oral food intake was longer in responders than in non-responders with a *p*-value < 0.001: cumulative incidence was 19.0 and 57.3% at 6 months, and 39.7 and 82.6% at 1-year, respectively.

**Additional file 3: Supplement Table 1.** The regimens administered after duodenal stenting and the indication of treatment lines.

## Data Availability

The datasets analysed during the current study are available from the corresponding author on reasonable request**.**

## Abbreviations

MGOO: Malignant gastric outlet obstruction
DS: duodenal stenting
TTSD: Time to stent dysfunction
ECOG PS: Eastern Cooperative Oncology Group performance status
