# Supplementary figures and images for: Association between time to stent dysfunction and the anti-tumour effect of systemic chemotherapy following stent placement in patients with pancreaticobiliary cancers and malignant gastric outlet obstruction: a retrospective cohort study
Source: BMC Cancer. 2021 May 19;21:576. doi: 10.1186/s12885-021-08336-z (PMC8136227; doi:10.1186/s12885-021-08336-z)

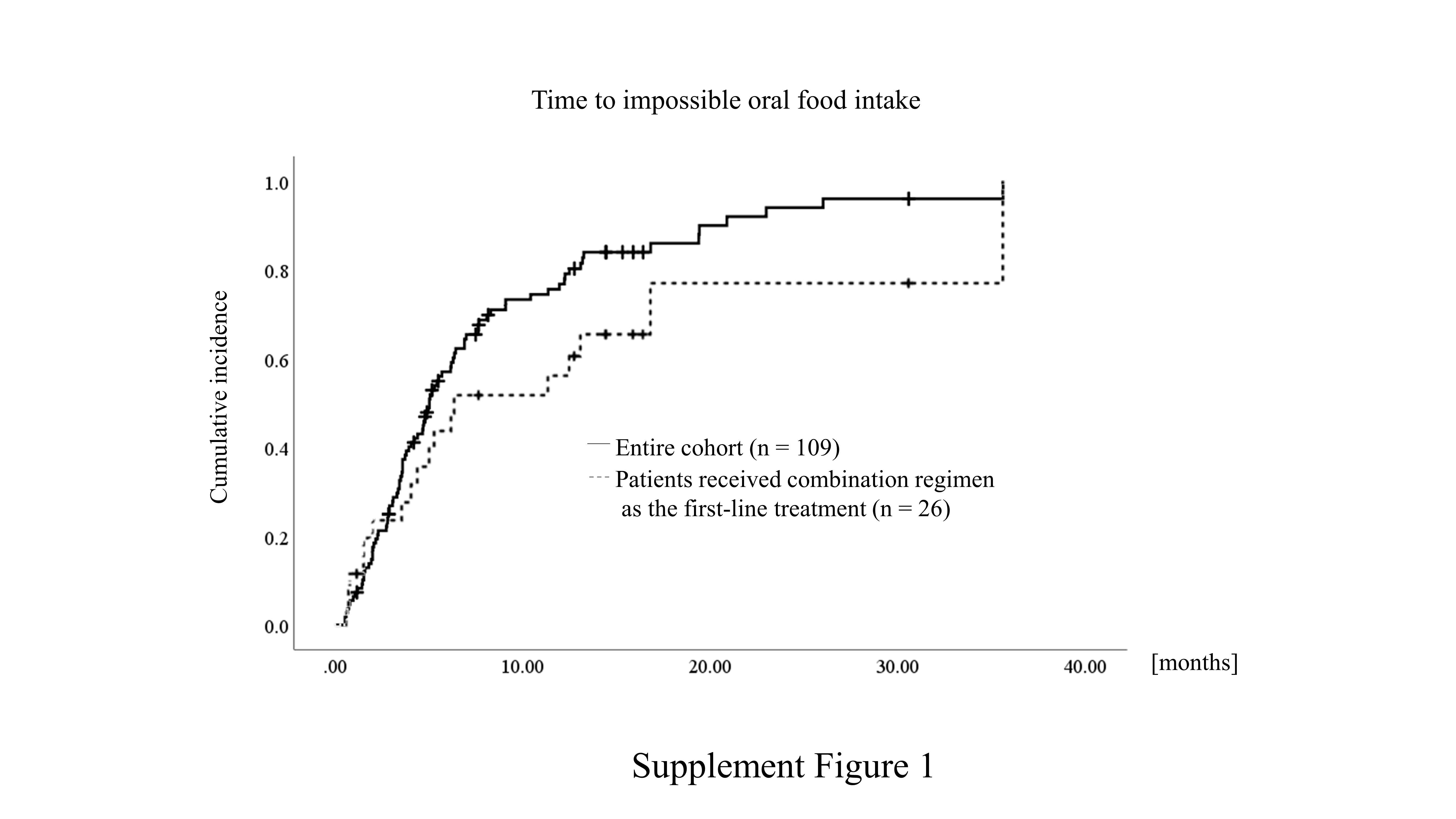

Supplement: Supplementary file 1 — Additional file 1: Supplement Figure 1. Time to impossible oral food intake in the entire cohort (solid line) and patients who received combination regimens as the first-line treatment (dotted line). Cumulative incidence of impossible oral food intake at 6-months and 1-year was 55.0 and 71.6% in the entire cohort, and 42.3 and 53.8% in patients who received combination regimens as the first-line treatment, respectively. [file 12885_2021_8336_MOESM1_ESM.tif]

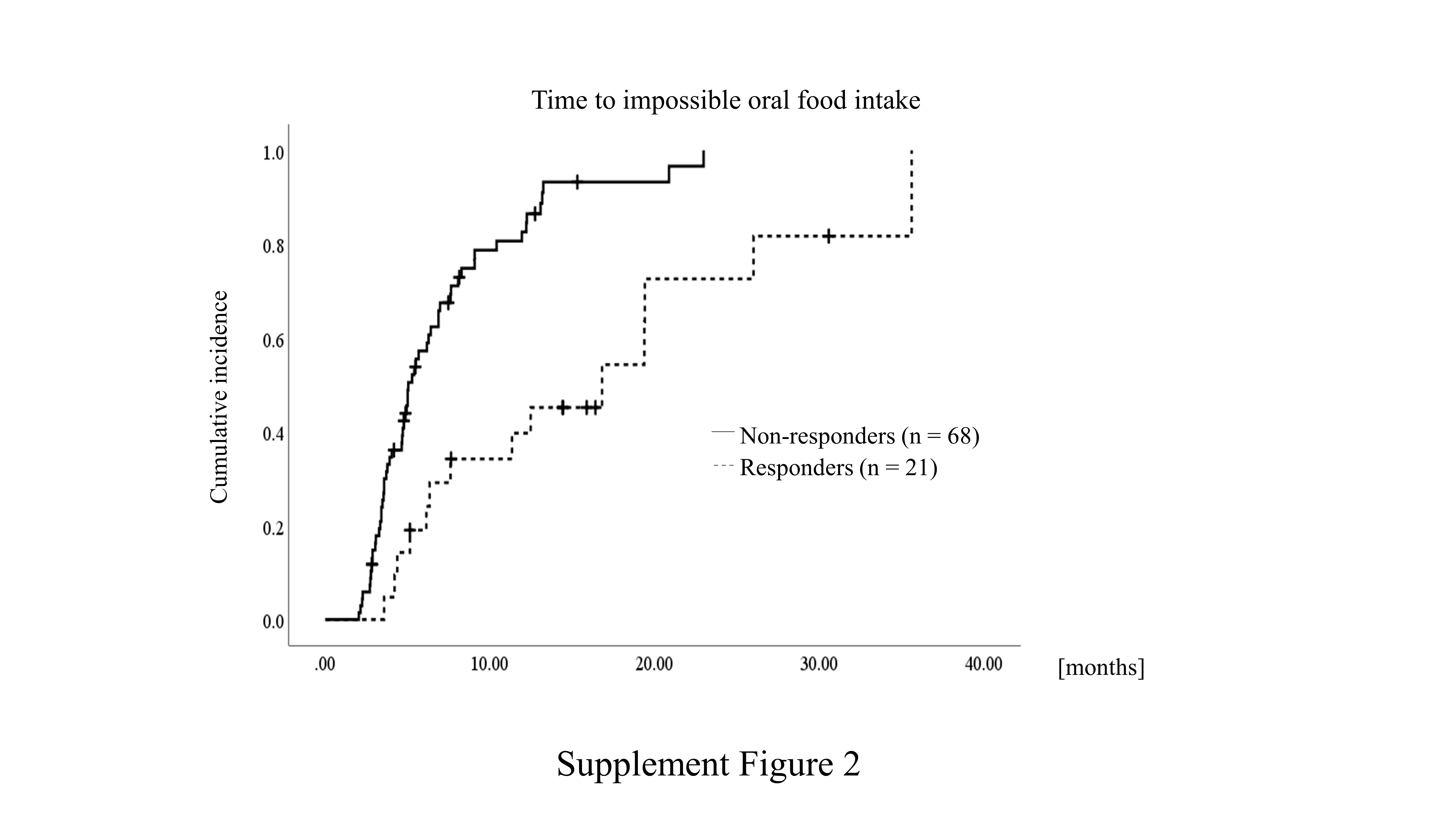

Supplement: Supplementary file 2 — Additional file 2: Supplement Figure 2. Time to impossible oral intake food in responders (dotted line) and non-responders (solid line) among patients who received combination regimen as the first-line treatment. Time to impossible oral food intake was longer in responders than in non-responders with a p-value < 0.001: cumulative incidence was 19.0 and 57.3% at 6 months, and 39.7 and 82.6% at 1-year, respectively. [file 12885_2021_8336_MOESM2_ESM.tif]
