# Supplementary material for: Association between time to stent dysfunction and the anti-tumour effect of systemic chemotherapy following stent placement in patients with pancreaticobiliary cancers and malignant gastric outlet obstruction: a retrospective cohort study
Source: BMC Cancer. 2021 May 19;21:576. doi: 10.1186/s12885-021-08336-z (PMC8136227; doi:10.1186/s12885-021-08336-z)
Supplement: Supplementary file 3 — Additional file 3: Supplement Table 1. The regimens administered after duodenal stenting and the indication of treatment lines. [file 12885_2021_8336_MOESM3_ESM.docx]

Supplement Table 1. The regimens administered after duodenal stenting and the indication of treatment lines

|  | Indication of treatment line | Regimen | Number of patients |
| --- | --- | --- | --- |
| Combination regimens | First-line | Gemcitabine + nab-paclitaxel | 22 |
|  |  | FOLFIRINOX | 2 |
|  |  | FOLFOX | 1 |
|  |  | Gemcitabine + Cisplatin | 1 |
|  | Second-fourth line | FOLFIRINOX | 8 |
|  |  | Gemcitabine + S-1 | 5 |
|  |  | Gemcitabine + nab-paclitaxel | 2 |
|  |  | FOLFIRI | 2 |
|  |  | FOLFOX | 1 |
|  |  | Gemcitabine + Cisplatin | 1 |
|  |  | Streptozocin + Fluorouacil | 1 |
| Monotherapy regimens | First-line | Gemcitabine | 19 |
|  |  | S-1 | 2 |
|  | Second-third line | S-1 | 32 |
|  |  | Gemcitabine | 5 |
|  |  | Streptozocin | 1 |
|  |  | Amrubicin | 1 |
|  |  | Everolimus | 1 |
|  |  | Investigational drug | 2 |

FOLFIRINOX, Fluorouracil, Leucovorin, Irinotecan and Oxaliplatin; FOLFIRI, Fluorouracil, Leucovorin and Irinotecan; FOLFOX, Fluorouracil, Leucovorin and Oxaliplatin
